# Mechanisms of Oxidized LDL-Mediated Endothelial Dysfunction and Its Consequences for the Development of Atherosclerosis

**DOI:** 10.3389/fcvm.2022.925923

**Published:** 2022-06-01

**Authors:** Hui Jiang, Yongwen Zhou, Seyed M. Nabavi, Amirhossein Sahebkar, Peter J. Little, Suowen Xu, Jianping Weng, Jianjun Ge

**Affiliations:** ^1^Department of Cardiothoracic Surgery, The First Affiliated Hospital of USTC, Division of Life Sciences and Medicine, University of Science and Technology of China, Hefei, China; ^2^Department of Endocrinology, Institute of Endocrine and Metabolic Diseases, The First Affiliated Hospital of USTC, Division of Life Sciences and Medicine, Clinical Research Hospital of Chinese Academy of Sciences (Hefei), University of Science and Technology of China, Hefei, China; ^3^Advanced Medical Pharma (BIOTEC), Benevento, Italy; ^4^Biotechnology Research Center, Pharmaceutical Technology Institute, Mashhad University of Medical Sciences, Mashhad, Iran; ^5^Applied Biomedical Research Center, Mashhad University of Medical Sciences, Mashhad, Iran; ^6^School of Health and Behavioural Sciences, Sunshine Coast Health Institute, University of the Sunshine Coast, Birtinya, QLD, Australia

**Keywords:** oxidized LDL, endothelial dysfunction, atherosclerosis, inflammation, oxidation

## Abstract

Atherosclerosis is an immuno-metabolic disease involving chronic inflammation, oxidative stress, epigenetics, and metabolic dysfunction. There is compelling evidence suggesting numerous modifications including the change of the size, density, and biochemical properties in the low-density lipoprotein (LDL) within the vascular wall. These modifications of LDL, in addition to LDL transcytosis and retention, contribute to the initiation, development and clinical consequences of atherosclerosis. Among different atherogenic modifications of LDL, oxidation represents a primary modification. A series of pathophysiological changes caused by oxidized LDL (oxLDL) enhance the formation of foam cells and atherosclerotic plaques. OxLDL also promotes the development of fatty streaks and atherogenesis through induction of endothelial dysfunction, formation of foam cells, monocyte chemotaxis, proliferation and migration of SMCs, and platelet activation, which culminate in plaque instability and ultimately rupture. This article provides a concise review of the formation of oxLDL, enzymes mediating LDL oxidation, and the receptors and pro-atherogenic signaling pathways of oxLDL in vascular cells. The review also explores how oxLDL functions in different stages of endothelial dysfunction and atherosclerosis. Future targeted pathways and therapies aiming at reducing LDL oxidation and/or lowering oxLDL levels and oxLDL-mediated pro-inflammatory responses are also discussed.

## Introduction

Atherosclerotic cardiovascular disease (ASCVD), the underlying pathophysiological condition which presents as cardio- and cerebro-vascular complications including ischemic stroke and myocardial infarction, is the major cause of death and disability worldwide (1). Oxidative stress, arises from multiple mechanisms including environmental pollution, unhealthy lifestyle factors such as cigarette smoking and physical inactivity and precipitating pathological conditions such as hypertension, hyperlipidemia, hyperuricemia, hyper-homocysteinemia and the hyperglycemia of diabetes, which has been proved to be the contributors or risk factors for the pathogenesis of CVD (2). The early event in atherosclerosis is the infiltration of low-density lipoproteins into the blood vessel wall where an inflammatory response is initiated and expressed as the leukocyte (mostly monocytes) penetration into the endothelium, the uptake of lipids and the formation of oxidized LDL (oxLDL) and a vicious cycle of chronic unresolving inflammation. These processes all contribute to the formation of the complex structures of plaques and the progression of the development of atherosclerosis (3).

The vascular endothelium plays a critical role in vascular quiescence and homeostasis. Analyzed from the perspective of cell biology and physiology, endothelium was originally described as a large, selectively permeable interface that separates the blood vessels and stroma of the body and acts as a gatekeeper, regulating the transport of fluid and macromolecules across vesicles and junctional complexes through a complex internal environment (4). Disturbances of the endothelium manifest as reduced production of nitric oxide (NO) or increased production of inflammatory cytokines and receptors is termed endothelial dysfunction and it appears to be a sine quo non for the occurrence of atherosclerosis. Endothelial cell dysfunction includes, in a broad sense, a series of non-adaptive changes in functional phenotype, hemostasis and thrombosis, local vascular tension and redox balance, and regulation of acute and chronic inflammation in the arterial wall (5). It is embodied in the focal infiltration, trapping and physicochemical modification of circulating lipoproteins under the sub-endothelium space (6). Endothelial cells in the endothelium are involved in multiple pathophysiological mechanisms including facilitating the inflammatory response, inflammasome activation, senescence, endothelial-mesenchymal transition (EndoMT), leukocyte adhesion, and cell death (2).

**Figure 1:**
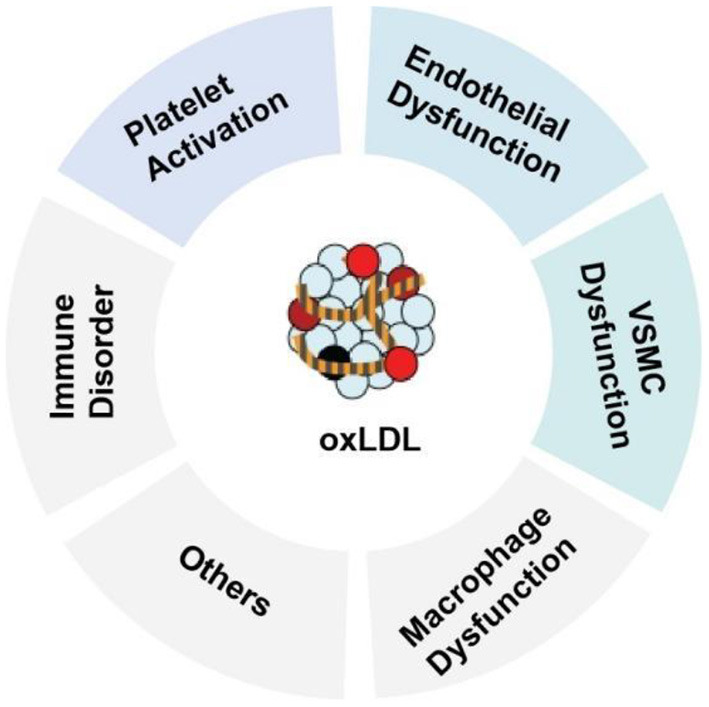
Role of oxidized LDL (oxLDL) in atherosclerosis.

oxLDL is one of the major factors leading to the activation, dysfunction, and injury of endothelial cells. Once the endothelium becomes dysfunctional, the vasodilatory properties of the vascular endothelium are impaired, leading to barrier disruption, increased vessel permeability, and increased expression of leukocyte adhesion molecules (2). Adherent monocytes will differentiate into macrophages in the subendothelial layer, where macrophages uptake oxLDL *via* the scavenger receptors [such as CD36, lectin-like oxLDL receptor-1 (LOX-1) and scavenger receptor A1] to transform the macrophages into lipid-laden foam cells (3). Uptake of oxLDL can also occurs in vascular smooth muscle cells (VSMCs), resulting in the emerging new type of foam cells—VSMC-derived foam cells. In addition, oxLDL can exert multiple effects on VSMCs, including stimulation of migration and proliferation, calcification, contractile-to-synthetic phenotypic switch and apoptosis (1). In macrophages, oxLDL and its lipotoxic components (such as lysophosphatidylcholine, LPC) can trigger inflammation, inflammasome activation and macrophage polarization. oxLDL can also promote platelet aggregation and activation (Figure 1). The persistent vascular inflammation, together with the impaired resolution of inflammation leads to plaque erosion, atherothrombosis, tissue ischemia and acute cardiovascular events (2). The majority of the atherogenic effects of oxLDL on endothelial function are regulated through the expression and activation of LOX-1(7). The multifactorial role of oxLDL in blood vessels makes it a prime candidate for exploring new disease mechanisms responsible for endothelial dysfunction and atherosclerosis and an ideal target for developing new cardiovascular drugs.

## Oxidative Stress and the Process of oxLDL Generation

**Figure 2:**
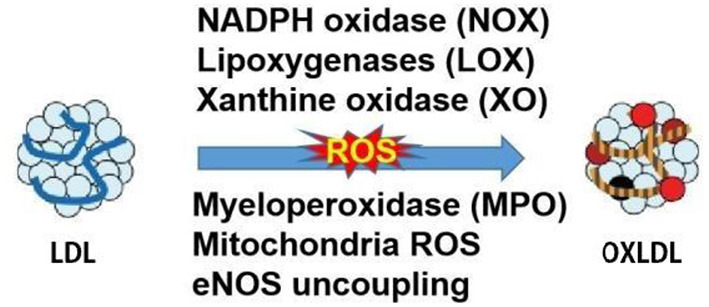
Enzymes that cause LDL oxidation.

Oxidative modifications of LDL have been postulated since the 1980s by Nobel prize recipients, Brown and Goldstein (8). Later in 1989 and 2002, the LDL oxidation was observed *in vivo* and oxLDL was identified in atherosclerotic lesions, rendering the study of LDL oxidation a necessary area of CVD research and highly clinically relevant (1). The LDL particles are composed of the hydrophobic core of polyunsaturated fatty acids, the esterified and unesterified cholesterol, and apolipoprotein-B. All components of LDL can be oxidized but the very small LDL was mostly prone to oxidation due to its easier penetration to the vascular wall. The oxidation of LDL is usually assumed to occur in the two main stages. Initially, the oxidation of LDL resulted in the little change of apolipoprotein B, which is called minimally oxidized LDL. Such modified LDL maintained the affinity to the LDL receptor and could induce inflammatory changes with increased chemokines and cytokines. With the redox homeostasis impaired by inflammation, the elevated levels of free radicals and the other oxidant categories originating from oxygen, nitrogen, and other chemical elements continued the oxidation of LDL. Subsequently, the modified LDL protein resulted in the shift of recognition from LDL receptor to the scavenger receptors, which accelerated the development of macrophage foam cells and the hallmark of artery lesion of fatty steak (9). *In vivo*, different layers of cultured vascular cells could be all damaged by the oxidized LDL and the first cell type that experiences the detrimental effects is the endothelial cells. Whereas, the endothelial and vascular smooth muscle cells could also catalyze the oxidation of LDL in turn. The relevant enzymes in the LDL oxidation are presented in Figure 2. LDL can be oxidized in different layers of cultured vascular cells. The relevant enzymes which are presented in Figure 2 include NADPH oxidase, lipoxygenases (LOX), xanthine oxidase (XO), myeloperoxidase (MPO), mitochondria reactive oxygen species (ROS) and uncoupled endothelial nitric oxide synthase (eNOS). MPO-oxidized LDL (Mox-LDL) is an important pathophysiological form for modified LDL *in vivo*. A very recent study has shown that Mox-LDL increases inflammation in macrophages through reducing the level of IL-10 (an anti-inflammatory cytokine) without affecting macrophage polarization (10). Elevated level of Mox-LDL will also potentially affect the functional status of endothelial cells and other types of vascular cells. At a practical level, in cell-free systems, oxLDL can be generated by incubation with Cu2+ but the extent of oxidation needs to be controlled and the LPS content in oxLDL preparations for experimental studies needs to be measured as a precautionary step to correctly ascribing actions to oxLDL.

The elimination of oxLDL from the circulation is through the phagocytes of reticuloendothelial system or *via* performed anti-oxLDL antibodies. Therefore, the strategy to prevent the atherosclerosis through the process of LDL oxidation is to lower the cholesterol load of lipoproteins, or to reduce the inflammation and oxidative stress. In this regard, the lipid-lowering drugs and many antioxidant compounds, including probucol and its analogs, vitamin E and C, coenzyme Q, phytochemicals such as the resveratrol, quercetin, probucol, tanshinone IIA, epigallocatechin gallate, and Lycopene, have been all reported to reduce LDL oxidation and atherosclerosis (11, 12). Clinical evidence of these antioxidants and the mechanism about inhibiting LDL oxidation had also been amply described in the recently published review (13).

## Cardiovascular Risk Factors That Increase oxLDL Level

Risk factors responsible for ROS production and LDL oxidation that contribute to atherosclerosis are well recognized (12). These risk factors include but are not limited to genetic predisposition, smoking, irradiation, infection, hypertension, diabetes/ hyperglycemia, obesity, insulin resistance, hyperlipidemia, hyperuricemia, hyper-homocysteinemia, environmental pollution (i.e., PM2.5), unhealthy diet and lifestyle factors such as physical inactivity (2). These factors are associated with a decrease of antioxidant enzymes (such as SOD, catalases and heme oxygenase 1) and the concomitant activation of NADPH oxidase system. These reactions lead to the excessive production of superoxide anions and eNOS uncoupling, the trigger of endothelial damage and apoptosis and lipid peroxidation, and further result in endothelial injury and dysfunction (1, 14).

## oxLDL and Endothelial Cell Dysfunction

### oxLDL Mediates Endothelial Cell Injury

The vascular endothelium serves as a homeostatic barrier between the circulating blood and the underlying components of the vascular wall. As the innermost layer of the vessel wall, the endothelial cells are usually susceptible to different atherogenic stimuli due to its direct exposure to the circulating blood. Furthermore, in response to the stimuli, a set of vasoactive molecules are usually secreted to regulate vascular tone through autocrine, endocrine and paracrine pathways, including nitric oxide (NO), prostacyclin (PGI2), endothelium-dependent hyperpolarization factor (EDRF), hydrogen sulfide (H2S), and endothelin-1 (ET-1) (2). One of the classical vascular endothelial cell injury models was established by treating endothelial cells from various vascular beds with oxLDL. In this model, the oxLDL is taken up by endothelial cells *via* oxLDL receptor 1 (LOX-1, also known as OLR1) (7, 15), leading to the activation of endothelial cell surface adhesion molecules expressed to trigger the adhesion of monocytes and their migration into sub-endothelial layers. And then the monocytes engulfed the oxLDL and transformed into macrophage-derived foam cells, which could further release a plethora of growth factors and thus promote the atherogenic process. There are multiple mechanisms resulting in the endothelial injury triggered by oxLDL, including the excessive ROS production, inflammation, and endoplasmic reticulum (ER) stress. In addition, oxLDL induced cytotoxicity can be reversed by several phytochemicals such as kaempferol, safflower yellow A, oxymatrine, and formononetin (16–18).

### Epigenetic Mechanisms Mediated Endothelial Injury—microRNA, LncRNA, and circRNA

Emerging evidence had also suggested that the endothelial injury is an epigenetic process with the interplay of multiple epigenetic mechanisms. The regulatory RNAs, such as microRNAs (miRNAs), long non-coding RNAs (lncRNAs) and circular RNAs (circRNAs), control the phenotype of blood vessels and immune cells and therefore play a vital role in atherosclerosis (19). As the detailed role of non-coding RNAs in atherosclerosis had been discussed elsewhere, here we will briefly summarize the role of the updated non-coding RNAs in atherosclerosis, which could help us further realize how many novel RNAs and how these novel RNAs affect the formation of lesions (20).

MicroRNAs are the endogenous non-coding small RNAs comprising 22–25 nucleotides, which are important in regulating gene expression by inhibiting transcription or promoting RNA degradation (21). Recently, the level of miR-328, miR345, miR-149, and miR-20a was reported to reduce dose-dependently in the model of endothelial injury induced by oxLDL. The miR-328, previously identified as crucial regulators in atherosclerosis, has been proved to protect the vascular endothelial cells against deleterious cellular events including cell viability, migration, invasion, apoptosis, and autophagy by targeting forkhead box protein O4 and the high-mobility group box 1 (HMGB1) (22, 23). The forced expression of miR-345 could markedly blocked apoptosis and inflammation in inhibiting the TAK1/p38/NF-kB pathway (24). For the miR-149, the alleviation from the oxLDL-induced endothelial injury was reported to through the promotion of autophagy *via* inhibiting the Akt/mTOR pathway while the vivo experiments are required to further investigate its potentials as the therapy (25). In addition, the miR-20a reduces the formation of the NLRP3 inflammasome through the thioredoxin-interacting protein (TXNIP) signaling and TLR4 and thus protects human aortic endothelial cells from oxLDL-induced injury (26). In contrast, there were also several miRNAs previously reported to increase in the oxLDL-induced model and thus the inhibition of these molecules might help protect from endothelial injury, including the miR-106 and miR214. With the dose-dependently increasing miR-106-5p observed in the oxLDL-induced model, the caspase-3 activity and ROS levels increased and STAT3 is proved to be the participated direct indicator (27). Similarly, the increasing expression of the miR-214-3p in oxLDL-induced injury resulted in the suppression of glutathione peroxidase 4 (GPX4) and the knockdown of miR-214 can partially reduce the ROS increase and restore the decreased expression of GPX4and eNOS (28).

Long non-coding RNA (LncRNAs, >200 nucleotides) have long been considered as “dark matter” of the human genome while with the functions increasingly reported in cardiovascular diseases, including the regulating of gene expression *via* transcriptional, post-transcriptional and epigenetic mechanisms, they had been exploited as useful biomarkers in recent years (19). Compared with miRNAs, lncRNAs have more diverse functions such as acting as chromatin regulators, guide, enhancers and sponge for miRNA to restrain the binding of miRNA and its target gene. More and more lncRNAs have been identified and functionally characterized in response to the stimuli of oxLDL and the mechanisms how these lncRNAs control gene expression and regulate the cellular function had been clarified. The increasing expression of small nucleolar RNA host gene 6 (SNHG6), observed in the oxLDL-induced model, had been reported recently to promote the progression of endothelial injury through the pathway mediated by miR-135a-5p and ROCK and the inhibition of SNHG6 help reduce the inflammation and oxidation stress (29). Similarly, the ZEB1-AS1 was also observed to participate in the endothelial injury induced by oxLDL with its increasing expression dose-independently and the further increasing levels of ROS and MDA, through the upregulating HMGB1 with the sponge of miR-942 and contributes to the apoptosis of endothelial cells (30). The exosome-mediated ZEB1-AS1 was also shown to facilitate cellular injury *via* signaling mediated with miR-590-5p and ETS1, and the pathway with TGF-β and Smad participated in oxLDL induced HUVECs (31). The inhibition of NEAT1 and the myocardial infarction-associated transcript (MIAT) expression in the oxLDL-induced model had also been reported to repress the endothelial dysfunction while their molecular pathways are quietly different. By promoting miR-30c-5p, the knockdown of NEAT1 could facilitate proliferation and repress apoptosis and inflammation in HUVECs treated with oxLDL *via* downregulating transcription factor 7 (32). As for MIAT, the increased expression of sponge of miR-206 was observed in the oxLDL-induced injury and the inhibition of miR-206 usually attenuated the protective effects from knockdown of MIAT by the accompanied promotion of Ras-related protein Rab-22A (33).

All of these deeper and broader understanding of lncRNAs provided us conceptual and mechanistic insights in the pathophysiology of atherosclerosis while there was still a long way to go for their development as the new therapeutic drugs. And for some lncRNAs, the reported effect on atherosclerosis is usually multiple. The recently reported LINC00657, also named as DNA damage activated non-coding RNA is an example. In the endothelial injury model induced by oxLDL, the expression of LINC00657 was increased while the silence of LINC00657 could result in the aggravation of cell senescence with the cell cycle arrest in G0/G1 phase, the increased oxidation stress with the increased ROS level, malondialdehyde and NF-κB nuclear translocation. In contrast, Wu H et al. observed that after knocking down the LINC00657 the inflammation and apoptosis could be inhibited *via* the miR-30c-5p/WNT7b/ β-catenin pathway, and thus inhibiting endothelial cell injury (34, 35). Therefore, the exact role of the LINC00657 in the endothelial injury has yet to be elucidated.

Circular RNA (CircRNA) is a highly conserved endogenous RNA that emerges as new regulators formed by non-sequential back-splicing of pre-mRNA transcripts. With the application of deep-scale sequencing, more and more circRNAs had been identified to function interactively with miRNAs, which affects the progression of atherosclerosis. The circ_0003204 (also called circ-USP36) has been demonstrated to serve as the sponge of several miRNAs and regulated the endothelial injury through different molecules recently. In the endothelial injury model induced by oxLDL, the levels of circ-USP36 usually increased. *Via* targeting the miR-330-5p and further upregulating the expression of TLR4, the NF-κB pathway would be activated to regulate the inflammation response, oxidase stress, and cell apoptosis (36). The levels of vascular cell adhesion molecule 1 (VCAM1) and roundabout guidance receptor 1(ROBO1) was also upregulated with the miR-98-5p and miR-20a-5p downregulated and sponged by circ-USP36 in oxLDL-induced injuries, which could both accelerate the endothelial cell injury (37, 38). However, differently, through absorbing the miR-637 to enhance WNT4, the circ-USP36 had been reported to attenuate the EC proliferation and migration in the oxLDL-induced injury (39). Due to the multiple targeted pathways, the epigenetic regulation network of circ-USP36 should be further discussed.

Another frequently discussed circRNA recently is the circRSF1. As reported, the circRSF1 expression was decreased after induction by oxLDL in HUVECs. Furthermore, serving as a molecular sponge, circRSF1 could negatively regulate both the miR-758 and miR-135-5p though the respective axis of cyclin D2 (CCDN2) and histone deacetylase 1, which could help damage the cell viability, cell migration, tube formation and thus finally endothelial injury (40, 41). Circ_0093887 was also validated to regulate cyclin D2 and succinate receptor 1. Serving as the sponge of miR-876-3p, the Circ_0093887 has also been reported to help protect HAECs against the oxLDL-induced inflammatory and apoptotic damages (39). Furthermore, for the circ_0068087, with similar targeted ROBO1 expression in circ-USP36, it not only aggravates hyperglycemia-induced dysfunction and inflammation of ECs with a sponge of miR-197 in diabetes but also alleviated oxLDL-induced dysfunction in HUVECs partly accompanied by reducing and upregulating miR-186-5p (42).

From the above evidences concluded, the role of non-coding RNAs in regulating multiple cellular functions involved in progression of the oxLDL-induced endothelial dysfunction is vital. As the epigenetic alternations are reversible, distinct from genetic mutations, these molecules are more accessible for modification and/or drug targeting and thus might be the promising therapeutical strategies in future.

### Severe Endothelial Injury Leads to Cell Death

When endothelial injury becomes severe, endothelial cells tend to experience different types of cell death (such as apoptosis, necrosis, pyroprosis, and ferroptosis), in particular, when endothelial cells are chronically exposed to various cardiovascular risk factors. For example, PCSK9 could be upregulated in HUVEC exposed to oxLDL and silencing its expression can inhibit the release of inflammatory substances (43). OxLDL-mediated oxidative stress has been shown to upregulate the expression of TXNIP which binds to NLRP3 and mediates the assembly and activation of NLRP3 inflammasome in endothelial cells exposed to high glucose. However, NLRP3-deficient HAECs were resistant to oxLDL-induced apoptotic cell death, maintained proliferative capacity, and reduced ROS production. Endothelial-specific depletion of NLRP3 also decrease apoptosis, cell death and ROS production (44).

In addition to genes in regulating endothelial cell death, non-coding RNAs also participate in endothelial cell apoptosis. Upon treatment with oxLDL, the expression of miR-30c-5p was increased, which inhibits NLRP3 inflammasome activation by targeting the FOXO3 (45). Upregulation of NLRP3 could also abrogate the protection of miR-223 (46). The fibroblast growth factor 21 (FGF21) is an endocrine cytokine which upregulates the ubiquinol cytochrome c reductase core protein I (UQCRC1) (47). Furthermore, melatonin, *via* upregulating UQCRC1, inhibits oxidative stress and cell pyroptosis *via* upregulating TET2, which might explain the anti-inflammatory and anti-atherogenic actions of melatonin (48).

Another key type of programmed cell death is ferroptosis. Ferroptosis was regulated by iron-dependent lipid peroxidation. Bai et al. discovered that the ferroptosis inhibitor Ferrostatin-1 (Fer-1) could alleviate iron accumulation and lipid peroxidation as well as the development of atherosclerotic lesions in ApoE^−/−^ mice (49). In addition, the SLC7A11 and GPX4, the two important anti-ferroptotic genes, were upregulated by Fer-1 treatment. In agreement with *in vivo* data, oxLDL-induced ferroptosis in MAECs was also reversed by Fer-1 and iron chelator desferrioxamine. This evidence suggests that strategies aimed at reducing oxLDL-induced endothelial cell death are hopeful to preserve endothelial viability and combat endothelial dysfunction.

### oxLDL Triggers Endothelial Inflammation and Leukocyte Adhesion

Once injured and becoming dead, endothelial cells lose barrier integrity which can lead to endothelial inflammation and accelerate leukocyte adhesion, rolling and transmigration (2). OxLDL is well-known trigger of leukocyte adhesion to endothelial cells (50). With the increase of relevant molecules (such as ICAM1, VCAM1, MCP-1, and E-selectin) mediating the cell rolling and adhesion, the leukocytes adhere to the endothelium and migrate into the intima. Then, the macrophages are activated, resulting in the release of proinflammatory cytokines accompanied by the production of ROS and proteolytic enzymes, contributing to the matrix degradation and plaque destabilization (1). The oxLDL and ensuing buildup of cholesterol crystals trigger the activation of NLRP3 inflammasome and fostering the vicious cycle of oxLDL/NLRP3 inflammasome/IL-1beta/pyroptosis (1).

A recent study has shown that oxLDL treatment leads to decreased expression of circ_0065149. However, overexpression of circ_0065149 leads to inhibition of NF-κB p65 subunit and the expression of IL-6 and IL-1β (51). These evidences suggest that genetic or epigenetic regulation of endothelial inflammation represents a promising way to attenuate endothelial dysfunction and atherosclerosis with several large-scale clinical trials discussed the efficacy of anti-inflammatory therapies in cardiovascular medicine, targeting IL-1β and IL-6.

### oxLDL Induces eNOS Uncoupling

In endothelial cells, eNOS is the main homeostatic enzyme which maintains the balance between NO and superoxide anion (O${}_{2}^{-}$) (52). The production of NO and the concentration of BH4 and GCH-1 was decreased after oxLDL treatment. The net effect is that oxLDL increased the production of superoxide anion and inhibited endothelium-dependent vasodilation. The activity of eNOS is positively regulated by the phosphorylation of Ser1177, while the Thr495 phosphorylation inhibits eNOS activation (53). In addition, related studies have shown that HSP90 can bind to eNOS, and induce its coupling and thus produce NO. Otherwise, the eNOS uncoupling, resulting in O${}_{2}^{-}$, would lead to impaired vasodilation (54, 55).

When cells are treated with oxLDL, the inhibition of eNOS could further aggravates oxLDL-induced p66^Shc^-mediated O2- production (56). The oxidation product of oxLDL, 1-palmitoyl-2-(5-oxovaleroyl)-sn-glycerol-3-phosphocholine (POVPC), is another important inflammatory lipid product in atherosclerosis. After incubation with POVPC, NO production in HUVECs is inhibited and apoptosis was increased evidenced by Bcl-2 inhibition and increased expression of Bax (57). There are several drugs and natural products that can affect eNOS uncoupling, such as ciglitazone. Ciglitazone-mediated PPARγ activation could suppress the LOX-1 expression and further reverse the oxLDL-induced decrease of eNOS and NO production. All these changes correlated with the AMPK/eNOS pathway (58). This evidence suggests that preventing oxLDL-induced eNOS uncoupling, is an alternative and complementary approach to ameliorate pro-atherogenic events elicited by oxLDL.

### oxLDL Induces EndoMT and Endothelial Dysfunction

Endothelial cells are heterogenous and highly plastic, which was evidenced by the transition of endothelial cells into mesenchymal-like cells (EndoMT) under pathological conditions (15). With the biotechnological advance of single-cell RNA-sequencing, the heterogeneity of endothelial cells in health and disease states can be described in more detail (59). The EndoMT is defined as the process of subsequent loss of endothelial cell morphology and function accompanied by phenotypic modulation and acquisition of the characteristics of mesenchymal cells occurring as increased capacity for proliferation, cell migration and extracellular matrix synthesis. Through EndoMT, endothelial cells lose their inter-cell contact and cell polarity, resulting in the molecular changes; consequences are calcification of plaque, the thinning of the fibrous cap, and plaque instability (60). Many triggering factors have been demonstrated to induce EndoMT and oxLDL represents one of the most triggering factors for EndoMT in the context of atherosclerosis (2).

Recent studies have revealed several new regulators in oxLDL-induced EndoMT. For example, interferon regulatory factor 2-binding protein 2, which attenuates macrophage-mediated inflammation and susceptibility to atherosclerosis, prevents the oxLDL-induced inflammation and EndoMT by upregulation of krüppel-like factor 2 (KLF2) (61). The compound-pyrogallol-phlprpglucinol-6,6-bieckol, has also been shown to attenuate oxLDL-induced EndoMT by increasing the expression of vWF, PECAM-1 and decreasing the expression of α-SMA and vimentin (62). In addition, LncRNA metastasis-associated lung adenocarcinoma transcript 1 (MALAT1) was upregulated in the oxLDL-treated HUVECs. Overexpression of MALAT1 could promote the activation of Wnt/β-catenin signaling pathway and reverse EndoMT (63). In the aortic tissues of ApoE^−/−^ mice and oxLDL-treated HUVECs, miR-200c-3p was highly expressed. miR-200c-3p functions by inhibiting SMAD7 and YAP expression (64). In addition, LncRNA ZFAS1 was shown to upregulate Notch3 expression *via* miR-150-5p. By doing so, the overexpression of ZFAS1 could promote the EndoMT in HUVECs (65). Therefore, based on the important role of EndoMT in atherosclerosis, the elucidation of novel targets in oxLDL triggered EndoMT will yield new therapeutic targets for cardiovascular diseases.

### oxLDL Induces Endothelial Cell Senescence

Endothelial senescence occurs due to the combinatorial mechanisms of oxidative stress, telomere shortening, inflammation, epigenetic modification, and longevity gene downregulation (66). OxLDL is a commonly used inducer of endothelial cell senescence. In this regard, ginsenoside Rb1 exerts protection against endothelial cell senescence through the SIRT1/Beclin-1/autophagy axis (67). In addition, GW9508, an agonist of GPR120, has been shown to improve oxLDL-induced cell cycle arrest. GW9508 also decreased the expression of p53 and plasminogen activator inhibitor 1, two standard hallmarks of senescent cells. Mechanistically, GW9508 promotes oxLDL-related translocation of NF-E2-related factor 2 (NRF2) to the nucleus (68). Similarly, quercetin treatment also reduces oxLDL-induced endothelial cell senescence in HAECs (69). In addition to oxLDL, a subfraction of oxLDL might also cause endothelial senescence, electronegative subfraction L5 in particular. L5 could lead to endothelial cell senescence by increasing nuclear γ-H2AX, the phosphorylation of Chk2, and p53 stabilization.

Numerous miRNAs also regulate endothelial senescence. The upregulation of miR-21-5p/203a-3p was observed in the oxLDL-induced HUVECs and hyperlipidemic rats. miR-21-5p/203a-3p could promote endothelial senescence by affecting the expression of dynamin related protein 1 (70). Based on these evidences, we can anticipate that more mechanisms of epigenetic regulation of endothelial cell senescence will be discovered, such as lncRNA and circular RNA-based mechanisms. Further elucidation of these epigenetic mechanisms will provide novel conceptual insights into the pathological mechanisms of endothelial cell senescence and yield novel senolytic drugs to abate endothelial dysfunction and atherosclerosis.

### oxLDL Triggers Endothelial Barrier Disruption and Hyperpermeability

The maintenance of barrier integrity of the endothelium is a major physiological function of endothelial cells. Barrier integrity is regulated by several microstructures on endothelial cells, such as the glycocalyx. Consisting of proteoglycans and glycoproteins, the endothelial glycocalyx binds many plasma proteins that are essential for vascular functioning (71). Clinically relevant concentrations of atherogenic oxLDL could reduce the thickness of the glycocalyx of endothelial cells, which effectively creates an interface for flowing red blood cell components, such as the platelets to the surface of vascular endothelial cells (72). Barrier disruption also leads to hyperpermeability in endothelial cells.

KLF2 is a crucial transcriptional factor downregulated by oxLDL (73). However, the novel anti-hypertensive agent, azilsartan, prevents oxLDL-induced endothelial monolayer hyperpermeability by increasing the expression of KLF2 and occludin. Interestingly, the protective effects of azilsartan were reversed by KLF2 silencing (51). Liraglutide, an anti-diabetic drug with cardiovascular protective actions could preserve endothelial barrier integrity by reversing oxLDL-induced downregulation of tight junctions' protein (74). These data demonstrate that agents that boost the expression of tight junction proteins could attenuate oxLDL-induced endothelial permeability.

## Conclusions and Perspectives

**Figure 3:**
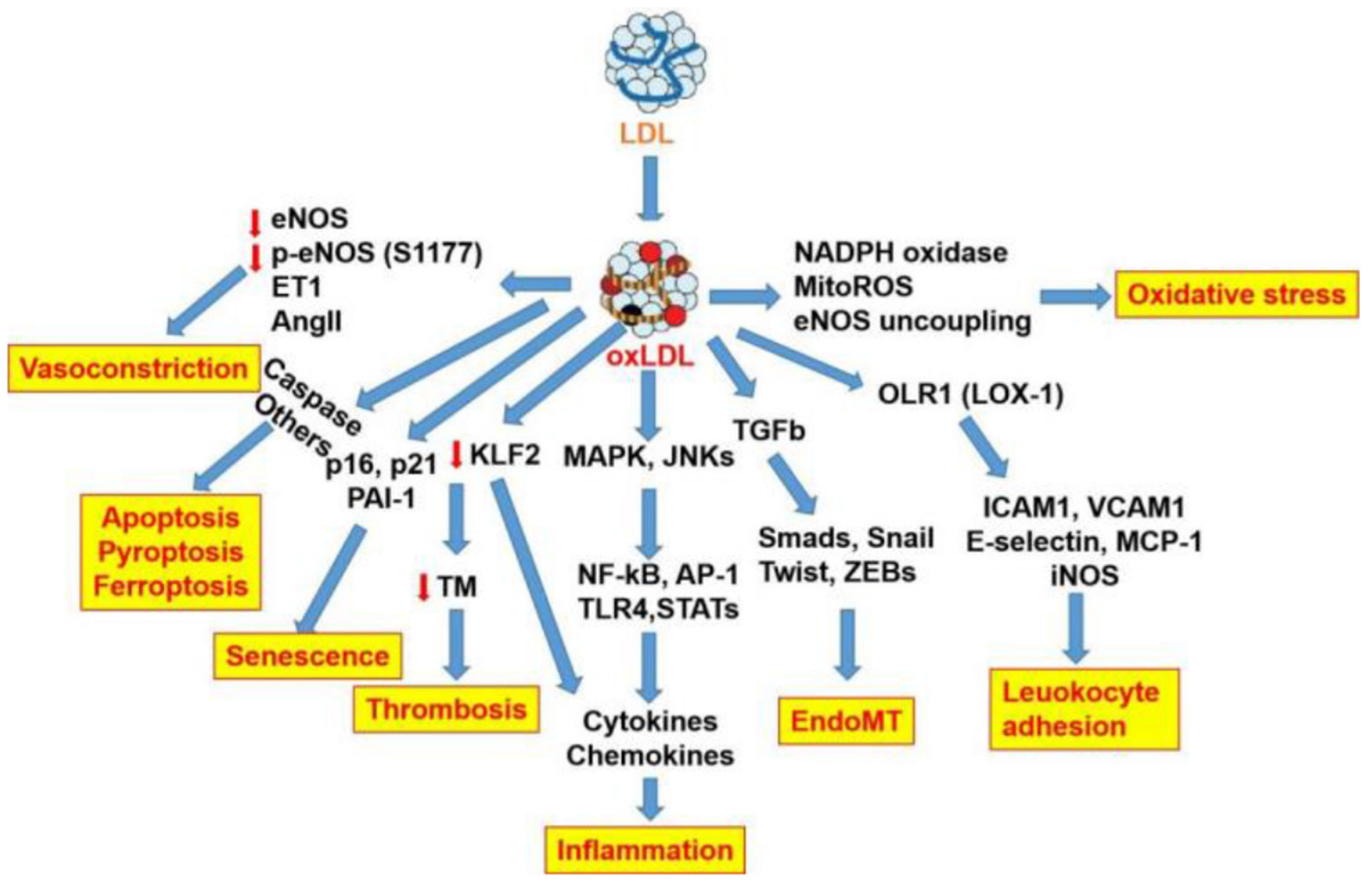
Endothelial pro-atherogenic events and signaling pathways mediated by oxLDL.

**Figure 4:**
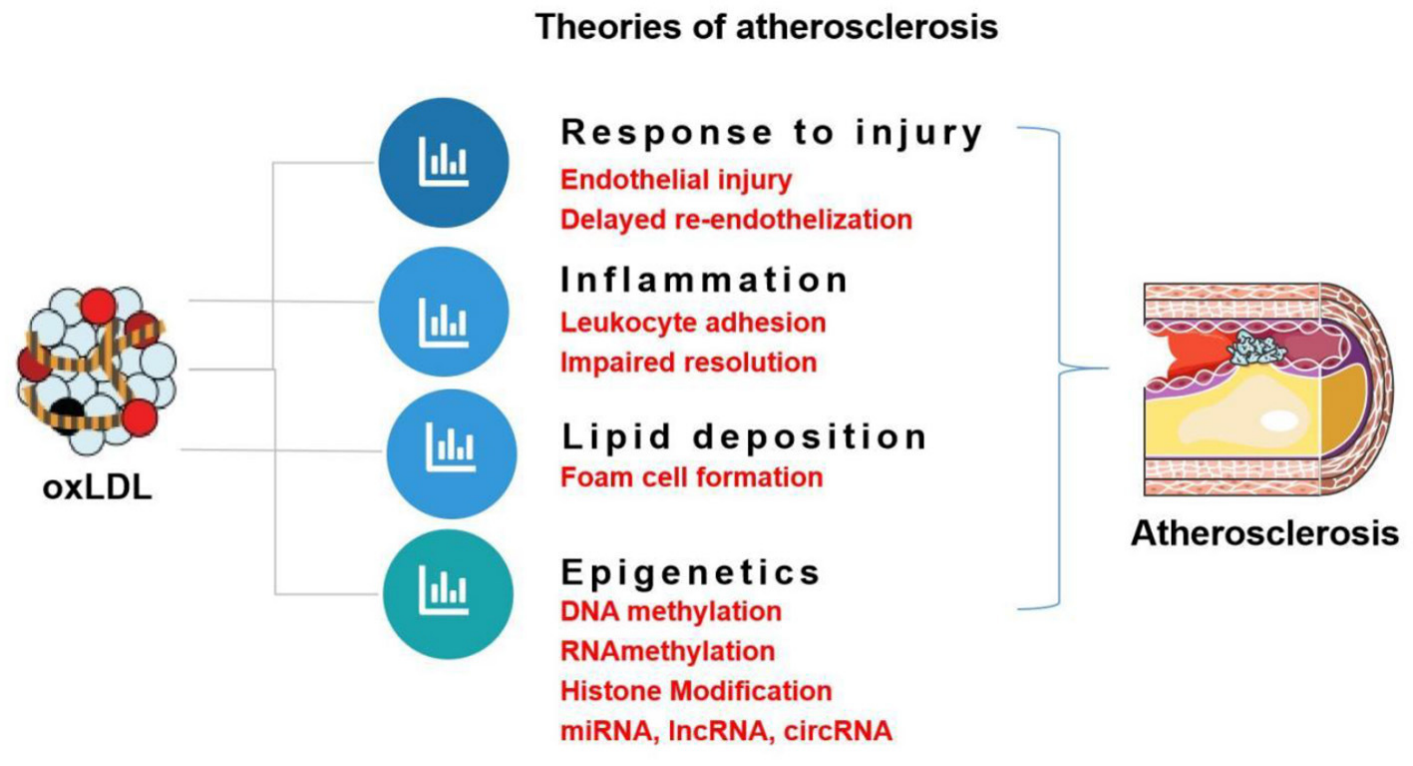
Pivotal role of oxLDL in main theories of atherosclerosis.

The role of oxLDL in atherogenic events and endothelial dysfunction as well as the respective signaling pathways is summarized in Figure 3. This review focuses on the role of oxLDL in endothelial dysfunction. Given that LDL oxidation represents one of the most prominent forms of atherogenic modifications and considering the existence of oxLDL in human atherosclerotic plaques, oxLDL serves as a pro-atherogenic stimulus for triggering endothelial dysfunction and mediating vascular disorders. OxLDL represents the main culprit in current theories of atherosclerosis, including “response to injury” theory, “Inflammation” theory, “lipid deposition” theory, and “epigenetic” theory (Figure 4). OxLDL has been suggested for many decades to trigger multiple aspects of endothelial dysfunction. Researchers in the past decades have proposed that oxLDL might serve as a promising therapeutic target for endothelial dysfunction. Further understanding of its atherogenic role and signaling pathways involved will lead to the discovery of novel therapeutic targets to prevent or treat cardiovascular diseases. Systems biology-based technologies, such as transcriptomic profiling of oxLDL-treated endothelial cells will elucidate new therapeutic modulators (such as oxLDL-specific and atherosclerosis-relevant miRNA, lncRNA and circular RNA) of endothelial function (75). Another possible mechanism for oxLDL-induced atherosclerosis may be exerted through crosstalk between endothelial cells and other types of cells *via* exosomal-dependent mechanisms. Future directions of oxLDL-related research can be summarized as follows.

### oxLDL-Mediated Epigenetic Regulation and Epigenetic Memory Effect

Emerging evidence has shown that oxLDL and other atherogenic stimuli also regulate the expression of epigenetic enzymes, such as DNMTs (19), TET2 (76), lncRNAs (77), and miRNAs (78) in endothelial cells. These new epigenetic mechanisms of oxLDL add additional layers of endothelial gene expression alteration by oxLDL. Detailed analysis of pathways involved will pave the way for new cardiovascular drug development. Notably, a high level of oxLDL in patients with CVD may exert epigenetic memory on endothelia cells even after lifestyle or pharmaceutical intervention to lower oxLDL levels. oxLDL can induce trained immunity in endothelial cells *via* metabolic and epigenetic reprogramming and is responsible for inflammation responses (79). The epigenetic memory effects of oxLDL could partially explain the residual cardiovascular risk in patients receiving standard care using lipid-lowering drugs, such as statins.

### High-Throughput Screening of Compounds Reducing LDL Oxidation and oxLDL Uptake

A large number of animal studies had supported oxLDL participate causally in atherosclerosis and the antioxidant drugs are assumed to be effective to prevent the atherosclerosis. There were also somewhat encouraging findings from observational studies observed to support this assumption. Yet, in the past 20 years, the results of the randomized clinical trials that evaluated the effect of antioxidants is disappointing, especially those for primary prevention. There were no overarching data to indicate that these antioxidant intervention work (80). Whether the choice of the antioxidants, the assessed dosage and the follow-up duration is rationale warrants further consideration (81). Nowadays, the biotechnological advances such as high-content and high-throughput compound screening has significantly accelerated new drug discovery. By these new technologies, new antioxidants or oxLDL-lowering drugs will be identified. Moreover, high-content screening of drug candidates which reduce oxLDL uptake by endothelial cells (by using DiI-labeled oxLDL) is a useful strategy to limit endothelial dysfunction and atherosclerosis. In addition to reducing LDL oxidation and oxLDL uptake, more attention should be focused the elucidation of novel therapies that target oxLDL-mediated pro-atherogenic signaling pathways.

### oxLDL on Endothelial Cell Metabolism and Trained Immunity

Emerging evidence has shown that endothelial cell metabolism represents an important component in cardiovascular physiology and pathology (2). As one of the most important pro-atherogenic stimuli, the potential role of oxLDL in endothelial cell metabolism, in particular glycolysis, remains to be evaluated because most energy sources (ATP) in endothelial cells arise from glycolysis rather than oxidative phosphorylation (82). A recent study has also shown that oxLDL-induced trained immunity in primary human monocyte-derived macrophages is dependent on oxidative phosphorylation activity (83). Endothelial cells have emerged as a new type of innate immune cells that fulfill immune regulatory functions. The role of oxLDL on trained immunity in endothelial cells remains to be evaluated by evidencing increased cytokine hyperresponsiveness to a second inflammation-related trigger. Moreover, the innate immune regulatory functions of endothelial cells to protect against oxLDL-induced atherogenic events remain to be identified.

### Discovery of oxLDL-Targeted Therapies

In light of the multiple pro-atherogenic role of oxLDL in triggering endothelial dysfunction and ASCVD, drugs or therapeutic agents that prevent LDL oxidation or reduce the level of oxLDL are presumably to attenuate endothelial dysfunction and ASCVD. In this regard, statins, liraglutide, azilsartan, antioxidants, and proprotein convertase subtilisin/kexin type 9 (PCSK9), have been demonstrated to reduce oxLDL level and delaying atherosclerosis as we have discussed above.

## Author Contributions

SX, JG, and JW: conceptualization and design. HJ, YZ, and SX: writing—original draft preparation. SN, AS, and PL: writing—review and editing. All authors have read and agreed to the published version of the manuscript.

## Funding

This study was supported by grants from National Key R&D Program of China (No.2021YFC2500500), National Natural Science Foundation of China (Grant Nos. 81941022, 81530025, and 82070464) and Strategic Priority Research Program of Chinese Academy of Sciences (Grant No. XDB38010100). This work was also supported by Program for Innovative Research Team of The First Affiliated Hospital of USTC (CXGG02), Anhui Provincial Key Research and Development Program (Grant No. 202104j07020051), Local Innovative and Research Teams Project of Guangdong Pearl River Talents Program (Grant No. 2017BT01S131), Hefei Comprehensive National Science Center (Grant No. BJ9100000005), and Hefei Municipal Development and Reform Commission Emergency Funding for COVID-19 disease. This work was also supported by the National Natural Science Foundation of China (grant numbers NSFC 81470530 to JG), Natural Science Foundation of Anhui Province (2008085MH240), and Major Science and Technology Project of Anhui Province (18030801132).

## Conflict of Interest

SN is employed by Advanced Medical Pharma (BIOTEC), Benevento, Italy. The remaining authors declare that the research was conducted in the absence of any commercial or financial relationships that could be construed as a potential conflict of interest.

## Publisher's Note

All claims expressed in this article are solely those of the authors and do not necessarily represent those of their affiliated organizations, or those of the publisher, the editors and the reviewers. Any product that may be evaluated in this article, or claim that may be made by its manufacturer, is not guaranteed or endorsed by the publisher.

## References

[1] Poznyak AV Nikiforov NG Markin AM Kashirskikh DA Myasoedova VA Gerasimova EV. Overview of OxLDL and its impact on cardiovascular health: focus on atherosclerosis. Front Pharmacol. (2020) 11:613780. 10.3389/fphar.2020.613780 33510639 PMC7836017

[2] Xu S Ilyas I Little PJ Li H Kamato D Zheng X. Endothelial dysfunction in atherosclerotic cardiovascular diseases and beyond: from mechanism to pharmacotherapies. Pharmacol Rev. (2021) 73:924–67. 10.1124/pharmrev.120.000096 34088867

[3] Wang D Yang Y Lei Y Tzvetkov NT Liu X Yeung AWK. Targeting foam cell formation in atherosclerosis: therapeutic potential of natural products. Pharmacol Rev. (2019) 71:596–670. 10.1124/pr.118.017178 31554644

[4] Karnovsky MJ. The ultrastructural basis of capillary permeability studied with peroxidase as a tracer. J Cell Biol. (1967) 35:213–36. 10.1083/jcb.35.1.213 6061717 PMC2107108

[5] Gimbrone MA García-Cardeña G. Endothelial cell dysfunction and the pathobiology of atherosclerosis. Circ Res. (2016) 118:620–36. 10.1161/CIRCRESAHA.115.306301 26892962 PMC4762052

[6] Simionescu N Vasile E Lupu F Popescu G Simionescu M. Prelesional events in atherogenesis. Accumulation of extracellular cholesterol-rich liposomes in the arterial intima and cardiac valves of the hyperlipidemic rabbit. Am J Pathol. (1986) 123:109–25. 3963146 PMC1888161

[7] Tian K Ogura S Little PJ Xu SW Sawamura T. Targeting LOX-1 in atherosclerosis and vasculopathy: current knowledge and future perspectives. Ann N Y Acad Sci. (2019) 1443:34–53. 10.1111/nyas.13984 30381837

[8] Brown MS Goldstein JL. Lipoprotein metabolism in the macrophage: implications for cholesterol deposition in atherosclerosis. Annu Rev Biochem. (1983) 52:223–61. 10.1146/annurev.bi.52.070183.001255 6311077

[9] Sacks D Baxter B Campbell BCV Carpenter JS Cognard C Dippel D. Multisociety consensus quality improvement revised consensus statement for endovascular therapy of acute ischemic stroke. Int J Stroke. (2018) 13:612–32. 10.1016/j.jvir.2017.11.026 29786478

[10] Bazzi S Frangie C Azar E Daher J. The effect of myeloperoxidase-oxidized LDL on THP-1 macrophage polarization and repolarization. Innate Immun. (2022) 28:91–103. 10.1177/17534259221090679 35404154 PMC9058374

[11] Dugas TR Morel DW Harrison EH. Impact of LDL carotenoid and alpha-tocopherol content on LDL oxidation by endothelial cells in culture. J Lipid Res. (1998) 39:999–1007. 10.1016/S0022-2275(20)33867-0 9610766

[12] Jialal I Fuller CJ. Effect of vitamin E, vitamin C and beta-carotene on LDL oxidation and atherosclerosis. Can J Cardiol. (1995) 11:97g−103g. 7585302

[13] Zhang S Li L Chen W Xu S Feng X Zhang L. Natural products: the role and mechanism in low-density lipoprotein oxidation and atherosclerosis. Phytother Res. (2021) 35:2945–67. 10.1002/ptr.7002 33368763

[14] Hu T Zhu P Liu Y Zhu H Geng J Wang B. PM2.5 induces endothelial dysfunction *via* activating NLRP3 inflammasome. Environ Toxicol. (2021) 36:1886–93. 10.1002/tox.23309 34173703

[15] Xu S Ogura S Chen J Little PJ Moss J Liu P. LOX-1 in atherosclerosis: biological functions and pharmacological modifiers. Cell Mol Life Sci. (2013) 70:2859–72. 10.1007/s00018-012-1194-z 23124189 PMC4142049

[16] Feng Z Wang C Yue J Meng Q Wu J Sun H. Kaempferol-induced GPER upregulation attenuates atherosclerosis *via* the PI3K/AKT/Nrf2 pathway. Pharm Biol. (2021) 59:1106–16. 10.1080/13880209.2021.1961823 34403325 PMC8436971

[17] Jin X Fu W Zhou J Shuai N Yang Y Wang B. Oxymatrine attenuates oxidized low-density lipoprotein-induced HUVEC injury by inhibiting NLRP3 inflammasome-mediated pyroptosis *via* the activation of the SIRT1/Nrf2 signaling pathway. Int J Mol Med. (2021) 48:187. 10.3892/ijmm.2021.5020 34368883 PMC8416146

[18] Zhang B Hao Z Zhou W Zhang S Sun M Li H. Formononetin protects against ox-LDL-induced endothelial dysfunction by activating PPAR-γ signaling based on network pharmacology and experimental validation. Bioengineered. (2021) 12:4887–98. 10.1080/21655979.2021.1959493 34369277 PMC8806800

[19] Xu S Kamato D Little PJ Nakagawa S Pelisek J Jin ZG. Targeting epigenetics and non-coding RNAs in atherosclerosis: from mechanisms to therapeutics. Pharmacol Ther. (2019) 196:15–43. 10.1016/j.pharmthera.2018.11.003 30439455 PMC6450782

[20] Schober A Maleki SS Nazari-Jahantigh M. Regulatory non-coding RNAs in atherosclerosis. Handb Exp Pharmacol. (2022) 270:463–92. 10.1007/164_2020_423 33454857

[21] Behrouz Sharif S Hashemzadeh S Mousavi Ardehaie R Eftekharsadat A Ghojazadeh M Mehrtash AH. Detection of aberrant methylated SEPT9 and NTRK3 genes in sporadic colorectal cancer patients as a potential diagnostic biomarker. Oncol Lett. (2016) 12:5335–43. 10.3892/ol.2016.5327 28105243 PMC5228494

[22] Qin X Guo J. MicroRNA-328-3p protects vascular endothelial cells against oxidized low-density lipoprotein induced injury *via* targeting forkhead box protein O4 (FOXO4) in atherosclerosis. Med Sci Monit. (2020) 26:e921877. 10.12659/MSM.921877 32329448 PMC7195608

[23] Wu C-Y Zhou Z-F Wang B Ke Z-P Ge Z-C Zhang X-J. MicroRNA-328 ameliorates oxidized low-density lipoprotein-induced endothelial cells injury through targeting HMGB1 in atherosclerosis. J Cell Biochem. (2019) 120:1643–50. 10.1002/jcb.27469 30324654

[24] Wei Q Tu Y Zuo L Zhao J Chang Z Zou Y. MiR-345-3p attenuates apoptosis and inflammation caused by oxidized low-density lipoprotein by targeting TRAF6 *via* TAK1/p38/NF-kB signaling in endothelial cells. Life Sci. (2020) 241:117142. 10.1016/j.lfs.2019.117142 31825793

[25] Zhu Z Li J Tong R Zhang X Yu B. miR-149 alleviates Ox-LDL-induced endothelial cell injury by promoting autophagy through Akt/mTOR pathway. Cardiol Res Pract. (2021) 2021:9963258. 10.1155/2021/9963258 34484820 PMC8416406

[26] Chen M Li W Zhang Y Yang J. MicroRNA-20a protects human aortic endothelial cells from Ox-LDL-induced inflammation through targeting TLR4 and TXNIP signaling. Biomed Pharmacother. (2018) 103:191–7. 10.1016/j.biopha.2018.03.129 29653364

[27] Hu Y Xu R He Y Zhao Z Mao X Lin L. Downregulation of microRNA106a5p alleviates oxLDLmediated endothelial cell injury by targeting STAT3. Mol Med Rep. (2020) 22:783–91. 10.3892/mmr.2020.11147 32626987 PMC7339537

[28] Xie M Huang P Wu T Chen L Guo R. Inhibition of miR-214-3p protects endothelial cells from ox-LDL-induced damage by targeting GPX4. Biomed Res Int. (2021) 2021:9919729. 10.1155/2021/9919729 34327240 PMC8277498

[29] Shan H Guo D Zhang S Qi H Liu S Du Y. SNHG6 modulates oxidized low-density lipoprotein-induced endothelial cells injury through miR-135a-5p/ROCK in atherosclerosis. Cell Biosci. (2020) 10:4. 10.1186/s13578-019-0371-2 31921409 PMC6947907

[30] Hua Z Ma K Liu S Yue Y Cao H Li Z. LncRNA ZEB1-AS1 facilitates ox-LDL-induced damage of HCtAEC cells and the oxidative stress and inflammatory events of THP-1 cells *via* miR-942/HMGB1 signaling. Life Sci. (2020) 247:117334. 10.1016/j.lfs.2020.117334 31962131

[31] Chen D Wang K Zheng Y Wang G Jiang M. Exosomes-mediated LncRNA ZEB1-AS1 facilitates cell injuries by miR-590-5p/ETS1 axis through the TGF-β/Smad pathway in oxidized low-density lipoprotein-induced human umbilical vein endothelial cells. J Cardiovasc Pharmacol. (2021) 77:480–90. 10.1097/FJC.0000000000000974 33818551

[32] Guo JT Wang L Yu HB. Knockdown of NEAT1 mitigates ox-LDL-induced injury in human umbilical vein endothelial cells *via* miR-30c-5p/TCF7 axis. Eur Rev Med Pharmacol Sci. (2020) 24:9633–44. 10.26355/eurrev_202009_23052 33015807

[33] Gao Y Yue J Huang Z. LncRNA MIAT mediates ox-LDL-induced endothelial cell injury *via* miR-206/RAB22A axis. J Surg Res. (2021) 265:303–12. 10.1016/j.jss.2021.02.029 33965771

[34] Bian W Jing X Yang Z Shi Z Chen R Xu A. Downregulation of LncRNA NORAD promotes Ox-LDL-induced vascular endothelial cell injury and atherosclerosis. Aging. (2020) 12:6385–400. 10.18632/aging.103034 32267831 PMC7185106

[35] Wu H Liu T Hou H. Knockdown of LINC00657 inhibits ox-LDL-induced endothelial cell injury by regulating miR-30c-5p/Wnt7b/beta-catenin. Mol Cell Biochem. (2020) 472:145–55. 10.1007/s11010-020-03793-9 32577947

[36] Su Q Dong X Tang C Wei X Hao Y Wu J. Knockdown of circ_0003204 alleviates oxidative low-density lipoprotein-induced human umbilical vein endothelial cells injury: Circulating RNAs could explain atherosclerosis disease progression. Open Med. (2021) 16:558–69. 10.1515/med-2021-0209 33869778 PMC8034243

[37] Peng K Jiang P Du Y Zeng D Zhao J Li M. Oxidized low-density lipoprotein accelerates the injury of endothelial cells *via* circ-USP36/miR-98-5p/VCAM1 axis. IUBMB Life. (2021) 73:177–87. 10.1002/iub.2419 33249762

[38] Miao J Wang B Shao R Wang Y. CircUSP36 knockdown alleviates oxidized low-density lipoprotein-induced cell injury and inflammatory responses in human umbilical vein endothelial cells *via* the miR-20a-5p/ROCK2 axis. Int J Mol Med. (2021) 47:40. 10.3892/ijmm.2021.4873 33576448 PMC7891832

[39] Huang JG Tang X Wang JJ Liu J Chen P Sun Y. A circular RNA, circUSP36, accelerates endothelial cell dysfunction in atherosclerosis by adsorbing miR-637 to enhance WNT4 expression. Bioengineered. (2021) 12:6759–70. 10.1080/21655979.2021.1964891 34519627 PMC8806706

[40] Wei Z Ran H Yang C. CircRSF1 contributes to endothelial cell growth, migration and tube formation under ox-LDL stress through regulating miR-758/CCND2 axis. Life Sci. (2020) 259:118241. 10.1016/j.lfs.2020.118241 32791147

[41] Zhang X Lu J Zhang Q Luo Q Liu B. CircRNA RSF1 regulated ox-LDL induced vascular endothelial cells proliferation, apoptosis and inflammation through modulating miR-135b-5p/HDAC1 axis in atherosclerosis. Biol Res. (2021) 54:11. 10.1186/s40659-021-00335-5 33757583 PMC7986494

[42] Li S Huang T Qin L Yin L. Circ_0068087 silencing ameliorates oxidized low-density lipoprotein-induced dysfunction in vascular endothelial cells depending on miR-186-5p-mediated regulation of roundabout guidance receptor 1. Front Cardiovasc Med. (2021) 8:650374. 10.3389/fcvm.2021.650374 34124191 PMC8187595

[43] Zeng J Tao J Xi L Wang Z Liu L. PCSK9 mediates the oxidative low-density lipoprotein-induced pyroptosis of vascular endothelial cells *via* the UQCRC1/ROS pathway. Int J Mol Med. (2021) 47:53. 10.3892/ijmm.2021.4886 33576442 PMC7895513

[44] Huang D Gao W Zhong X Ge J. NLRP3 activation in endothelia promotes development of diabetes-associated atherosclerosis. Aging. (2020) 12:18181–91. 10.18632/aging.103666 32966239 PMC7585081

[45] Li P Zhong X Li J Liu H Ma X He R. MicroRNA-30c-5p inhibits NLRP3 inflammasome-mediated endothelial cell pyroptosis through FOXO3 down-regulation in atherosclerosis. Biochem Biophys Res Commun. (2018) 503:2833–40. 10.1016/j.bbrc.2018.08.049 30119891

[46] Wang X Li X Wu Y Song Y. Upregulation of miR-223 abrogates NLRP3 inflammasome-mediated pyroptosis to attenuate oxidized low-density lipoprotein (ox-LDL)-induced cell death in human vascular endothelial cells (ECs). In Vitro Cell Dev Biol Anim. (2020) 56:670–9. 10.1007/s11626-020-00496-9 32914384

[47] Chen JJ Tao J Zhang XL Xia LZ Zeng JF Zhang H. Inhibition of the ox-LDL-induced pyroptosis by FGF21 of human umbilical vein endothelial cells through the TET2-UQCRC1-ROS pathway. DNA Cell Biol. (2020) 39:661–70. 10.1089/dna.2019.5151 32101022

[48] Zeng J Tao J Xia L Zeng Z Chen J Wang Z. Melatonin inhibits vascular endothelial cell pyroptosis by improving mitochondrial function *via* up-regulation and demethylation of UQCRC1. Biochem Cell Biol. (2021) 99:339–47. 10.1139/bcb-2020-0279 33332241

[49] Bai T Li M Liu Y Qiao Z Wang Z. Inhibition of ferroptosis alleviates atherosclerosis through attenuating lipid peroxidation and endothelial dysfunction in mouse aortic endothelial cell. Free Radic Biol Med. (2020) 160:92–102. 10.1016/j.freeradbiomed.2020.07.026 32768568

[50] Obermayer G Afonyushkin T Binder CJ. Oxidized low-density lipoprotein in inflammation-driven thrombosis. J Thromb Haemost. (2018) 16:418–28. 10.1111/jth.13925 29316215

[51] Li D Jin W Sun L Wu J Hu H Ma L. Circ_0065149 alleviates oxidized low-density lipoprotein-induced apoptosis and inflammation in atherosclerosis by targeting miR-330-5p. Front Genet. (2021) 12:590633. 10.3389/fgene.2021.590633 33603770 PMC7884639

[52] Förstermann U Sessa WC. Nitric oxide synthases: regulation and function. Eur Heart J. (2012) 33:829–37, 837a–d. 10.1093/eurheartj/ehr304 21890489 PMC3345541

[53] Das M Devi KP Belwal T Devkota HP Tewari D Sahebnasagh A. Harnessing polyphenol power by targeting eNOS for vascular diseases. Crit Rev Food Sci Nutr. (2021). 10.1080/10408398.2021.1971153. [Epub ahead of print]. 34553653

[54] Fujimura N Jitsuiki D Maruhashi T Mikami S Iwamoto Y Kajikawa M. Geranylgeranylacetone, heat shock protein 90/AMP-activated protein kinase/endothelial nitric oxide synthase/nitric oxide pathway, and endothelial function in humans. Arterioscler Thromb Vasc Biol. (2012) 32:153–60. 10.1161/ATVBAHA.111.237263 21998134

[55] Ning DS Ma J Peng YM Li Y Chen YT Li SX. Apolipoprotein A-I mimetic peptide inhibits atherosclerosis by increasing tetrahydrobiopterin *via* regulation of GTP-cyclohydrolase 1 and reducing uncoupled endothelial nitric oxide synthase activity. Atherosclerosis. (2021) 328:83–91. 10.1016/j.atherosclerosis.2021.05.019 34118596

[56] Shi Y LÜSCHER TF Camici GG. Dual role of endothelial nitric oxide synthase in oxidized LDL-induced, p66Shc-mediated oxidative stress in cultured human endothelial cells. PLoS ONE. (2014) 9:e107787. 10.1371/journal.pone.0107787 25247687 PMC4172699

[57] Yan FX Li HM Li SX He SH Dai WP Li Y. The oxidized phospholipid POVPC impairs endothelial function and vasodilation *via* uncoupling endothelial nitric oxide synthase. J Mol Cell Cardiol. (2017) 112:40–8. 10.1016/j.yjmcc.2017.08.016 28870504

[58] Xu L Wang S Li B Sun A Zou Y Ge J. A protective role of ciglitazone in ox-LDL-induced rat microvascular endothelial cells *via* modulating PPARγ-dependent AMPK/eNOS pathway. J Cell Mol Med. (2015) 19:92–102. 10.1111/jcmm.12463 25388834 PMC4288353

[59] Tombor LS John D Glaser SF Luxán G Forte E Furtado M. Single cell sequencing reveals endothelial plasticity with transient mesenchymal activation after myocardial infarction. Nat Commun. (2021) 12:681. 10.1038/s41467-021-20905-1 33514719 PMC7846794

[60] Evrard SM Lecce L Michelis KC Nomura-Kitabayashi A Pandey G Purushothaman KR. Endothelial to mesenchymal transition is common in atherosclerotic lesions and is associated with plaque instability. Nat Commun. (2016) 7:11853. 10.1038/ncomms11853 27340017 PMC4931033

[61] Jiang Y Shen Q. IRF2BP2 prevents ox-LDL-induced inflammation and EMT in endothelial cells via regulation of KLF2. Exp Ther Med. (2021) 21:481. 10.3892/etm.2021.9912 33767776 PMC7976449

[62] Son M Oh S Jang JT Son KH Byun K. Pyrogallol-phloroglucinol-6 6-bieckol on attenuates high-fat diet-induced hypertension by modulating endothelial-to-mesenchymal transition in the aorta of mice. Oxid Med Cell Longev. (2021) 2021:8869085. 10.1155/2021/8869085 33574986 PMC7857897

[63] Li H Zhao Q Chang L Wei C Bei H Yin Y. LncRNA MALAT1 modulates ox-LDL induced EndMT through the Wnt/β-catenin signaling pathway. Lipids Health Dis. (2019) 18:62. 10.1186/s12944-019-1006-7 30871555 PMC6417088

[64] Mao Y Jiang L. MiR-200c-3p promotes ox-LDL-induced endothelial to mesenchymal transition in human umbilical vein endothelial cells through SMAD7/YAP pathway. J Physiol Sci. (2021) 71:30. 10.1186/s12576-021-00815-z 34525946 PMC10717414

[65] Yin Q He M Huang L Zhang X Zhan J Hu J. lncRNA ZFAS1 promotes ox-LDL induced EndMT through miR-150-5p/Notch3 signaling axis. Microvasc Res. (2021) 134:104118. 10.1016/j.mvr.2020.104118 33278458

[66] Huang Y Hu C Ye H Luo R Fu X Li X. Inflamm-aging: a new mechanism affecting premature ovarian insufficiency. J Immunol Res. (2019) 2019:8069898. 10.1155/2019/8069898 30719458 PMC6334348

[67] Shi G Liu D Zhou B Liu Y Hao B Yu S. Ginsenoside Rb1 alleviates oxidative low-density lipoprotein-induced vascular endothelium senescence *via* the SIRT1/Beclin-1/autophagy axis. J Cardiovasc Pharmacol. (2020) 75:155–67. 10.1097/FJC.0000000000000775 31658172

[68] Liu R Cheng F Zeng K Li W Lan J. GPR120 agonist GW9508 ameliorated cellular senescence induced by ox-LDL. ACS Omega. (2020) 5:32195–202. 10.1021/acsomega.0c03581 33376857 PMC7758881

[69] Jiang YH Jiang LY Wang YC Ma DF Li X. Quercetin attenuates atherosclerosis via modulating oxidized LDL-induced endothelial cellular senescence. Front Pharmacol. (2020) 11:512. 10.3389/fphar.2020.00512 32410992 PMC7198817

[70] Zhang JJ Liu WQ Peng JJ Ma QL Peng J Luo XJ. miR-21-5p/203a-3p promote ox-LDL-induced endothelial cell senescence through down-regulation of mitochondrial fission protein Drp1. Mech Ageing Dev. (2017) 164:8–19. 10.1016/j.mad.2017.03.009 28347692

[71] Sörensson J Matejka GL Ohlson M Haraldsson B. Human endothelial cells produce orosomucoid, an important component of the capillary barrier. Am J Physiol. (1999) 276:H530–4. 10.1152/ajpheart.1999.276.2.H530 9950854

[72] Vink H Constantinescu AA Spaan JA. Oxidized lipoproteins degrade the endothelial surface layer : implications for platelet-endothelial cell adhesion. Circulation. (2000) 101:1500–2. 10.1161/01.CIR.101.13.1500 10747340

[73] Li W Wang C Zhang D Zeng K Xiao S Chen F. Azilsartan ameliorates ox-LDL-induced endothelial dysfunction *via* promoting the expression of KLF2. Aging. (2021) 13:12996–3005. 10.18632/aging.202973 33946046 PMC8148451

[74] Yue W Li Y Ou D Yang Q. The GLP-1 receptor agonist liraglutide protects against oxidized LDL-induced endothelial inflammation and dysfunction via KLF2. IUBMB Life. (2019) 71:1347–54. 10.1002/iub.2046 30969479

[75] Su D Yi L Guan L Li Q Shi C Ma X. Sequencing analysis of mRNA profile in endothelial cells in response to ox-LDL. Biochem Genet. (2021) 59:767–80. 10.1007/s10528-021-10028-z 33528699

[76] Peng J Tang ZH Ren Z He B Zeng Y Liu LS. TET2 Protects against oxLDL-Induced HUVEC Dysfunction by Upregulating the CSE/H(2)S System. Front Pharmacol. (2017) 8:486. 10.3389/fphar.2017.00486 28798687 PMC5526911

[77] Singh KK Matkar PN Pan Y Quan A Gupta V Teoh H. Endothelial long non-coding RNAs regulated by oxidized LDL. Mol Cell Biochem. (2017) 431:139–49. 10.1007/s11010-017-2984-2 28316063

[78] Khaidakov M Mitra S Wang X Ding Z Bora N Lyzogubov V. Large impact of low concentration oxidized LDL on angiogenic potential of human endothelial cells: a microarray study. PLoS ONE. (2012) 7:e47421. 10.1371/journal.pone.0047421 23115646 PMC3480370

[79] Drummer CT Saaoud F Shao Y Sun Y Xu K Lu Y. Trained immunity and reactivity of macrophages and endothelial cells. Arterioscler Thromb Vasc Biol. (2021) 41:1032–46. 10.1161/ATVBAHA.120.315452 33380171 PMC7904591

[80] Leopold JA. Antioxidants and coronary artery disease: from pathophysiology to preventive therapy. Coron Artery Dis. (2015) 26:176–83. 10.1097/MCA.0000000000000187 25369999 PMC4315737

[81] Libby P. The changing landscape of atherosclerosis. Nature. (2021) 592:524–33. 10.1038/s41586-021-03392-8 33883728

[82] De Bock K Georgiadou M Schoors S Kuchnio A Wong BW Cantelmo AR. Role of PFKFB3-driven glycolysis in vessel sprouting. Cell. (2013) 154:651–63. 10.1016/j.cell.2013.06.037 23911327

[83] Groh LA Ferreira AV Helder L Van Der Heijden C Novakovic B Van De Westerlo E. oxLDL-induced trained immunity is dependent on mitochondrial metabolic reprogramming. Immunometabolism. (2021) 3:e210025. 10.20900/immunometab20210025 34267957 PMC7611242

